# Ameliorative Effects of Pumpkin Seed Protein Peptides on Dexamethasone-Treated Sarcopenia and Their Effects When Combined with Vitamin D

**DOI:** 10.3390/foods15071162

**Published:** 2026-03-30

**Authors:** Donghui Ma, Yuxin Liu, Jing Zhao, Quanhong Li

**Affiliations:** 1 College of Food Science and Nutritional Engineering, China Agricultural University, Beijing 100083, China; 2 China National Engineering Research Center for Fruit & Vegetable Processing, Beijing 100083, China; 3 CAU-SCCD Advanced Agricultural & Industrial Institute, Chengdu 611400, China

**Keywords:** pumpkin seed protein peptides, sarcopenia, dexamethasone, C2C12

## Abstract

Sarcopenia is a degenerative condition that imposes a substantial global public health burden, yet safe and effective interventions remain limited. Nutritional support is regarded as an important strategy to mitigate age-related muscle loss and improve physical function in older adults. Due to time and cost constraints, dexamethasone (DEX)-treated models are often used as an alternative to age-related sarcopenia models. This study investigated the effects of pumpkin seed protein peptides (PSPP) and vitamin D on DEX-treated mice. In vitro, PSPP attenuated senescence-associated phenotypes, reduced cellular injury, and partially alleviated DEX-treated myofibrillar atrophy, as evidenced by decreased Atrogin-1 and MuRF1 expression and increased MyoD expression. In vivo, PSPP and vitamin D, particularly in combination, ameliorated DEX-treated declines in muscle mass, grip strength, and endurance. Histological analyses further demonstrated improvements in myofibrillar architecture and muscle fiber cross-sectional area. In addition, each intervention was associated with increased ATP content, elevated interleukin-10 and insulin-like growth factor-1 levels, and reduced tumor necrosis factor-α and malondialdehyde levels. Collectively, these findings suggest that PSPP, either alone or combined with vitamin D, may alleviate DEX-treated sarcopenia, potentially through the modulation of mitochondrial homeostasis, attenuation of oxidative stress and inflammatory responses, and promotion of myogenic regeneration.

## 1. Introduction

As life expectancy increases and population aging accelerates, age-related diseases, such as sarcopenia, are becoming major drivers of household healthcare burden and public health expenditure worldwide [1,2]. Sarcopenia, a syndrome characterized by age-related declines in muscle quantity, quality, strength, and function, is a major age-related condition. Sarcopenia can substantially impair mobility in older adults and increase the risk of falls, fractures, disability, and related adverse health outcomes [3]. A recent systematic review and meta-analysis of 41 studies involving 34,955 participants worldwide reported community-based sarcopenia prevalence rates of 11% in men and 9% in women [4]. Sarcopenia involves an imbalance between muscle protein synthesis and degradation and reflects a complex disorder characterized by dysregulated signaling pathways. It is commonly associated with aging, malnutrition, chronic inflammation, redox imbalance, skeletal muscle mitochondrial dysfunction, and altered hormonal status [5,6,7]. Notably, no pharmacological therapy has yet been approved for the treatment of sarcopenia. Therefore, there remains a clear need to identify safe, effective, and cost-effective approaches for the prevention and management of sarcopenia.

Although aerobic exercise, resistance training, and balance training can improve muscle strength in older adults with sarcopenia, defining a safe and effective training intensity remains challenging, and excessive loads may increase injury risk [8,9]. Therefore, non-exercise approaches, particularly nutrition and dietary supplementation, remain important strategies to counteract age-related muscle loss. Several countries, including China, the United States, and South Korea, recommend increased dietary protein intake as part of strategies to alleviate sarcopenia in older adults [10]. Adequate protein intake has been shown to stimulate muscle protein synthesis and reduce protein breakdown, thereby helping maintain muscle strength and physical function in older adults [11]. For example, whey protein, which is rich in branched-chain amino acids, has been reported to modulate gut microbiota composition in dexamethasone (DEX)-treated mice and to be associated with attenuated skeletal muscle atrophy [12,13]. Although the muscle-building benefits of dietary protein are well supported [14,15], aging may reduce small-intestinal absorptive capacity, thereby compromising protein digestion and decreasing amino acid availability for muscle protein synthesis [16]. Hydrolyzing dietary proteins into peptides is a practical approach to improve digestibility and absorption and may also enhance bioactivity. Accumulating evidence suggests that bioactive peptides may mitigate muscle atrophy through multiple mechanisms. For instance, corn peptides [17] and grouper peptides [18] have been reported to activate the PI3K/Akt/mTOR pathway, which may promote muscle protein synthesis and myofibrillar growth. Tilapia skin peptides [19] and mealworm peptides [20] have been reported to improve mitochondrial function via the SIRT1-PGC-1α pathway, potentially supporting energy metabolism and endurance in atrophy-related contexts. Chicken breast peptides [10] have been reported to increase short-chain fatty acid (SCFA) levels (e.g., acetate, propionate, and isovalerate), modulate gut microbiota composition, and potentially influence muscle metabolism via the gut-muscle axis. Additionally, wheat oligopeptides [16] have been linked to improved muscle function and fiber structure, potentially via antioxidant activity and attenuation of oxidative stress. Low-molecular-weight hemp (*Cannabis sativa* L.) seed peptides [21], which are rich in branched-chain amino acids, have been reported to protect against muscle atrophy, possibly due to improved absorption and upregulation of myogenesis-related proteins.

The amino acid profile of pumpkin seed protein meets the FAO/WHO recommended requirements for essential amino acids in adults. Moreover, it has been reported to exhibit bioactive properties, including antidiabetic, anticancer, and antioxidant activities [22]. Nevertheless, its limited solubility and emulsifying capacity limit its broader application in food systems [23]. Enzymatic hydrolysis has been shown to enhance these functional properties while preserving the protein’s nutritional integrity [24]. Previous studies also have suggested that pumpkin seed protein peptides (PSPP) exhibit antioxidant and anti-inflammatory activities [25]. However, their potential role in sarcopenia has not been investigated. Meanwhile, vitamin D supplementation has attracted sustained attention in the context of sarcopenia, yet reported outcomes remain inconsistent [26,27,28]. Here, we report a two-stage study integrating a DEX-treated C2C12 model and a mouse model to evaluate the effects of PSPP and vitamin D on sarcopenia and to explore potential underlying mechanisms. Together, our results suggest that co-intervention targeting protein turnover and mitochondrial function using PSPP and vitamin D may represent a promising strategy for the prevention and management of sarcopenia.

## 2. Materials and Methods

### 2.1. Materials

Pumpkin seed protein powder was purchased from BAk Bio-Tech Co., Ltd. (Baoji, China). Alcalase and flavor protease were purchased from Novozymes (Copenhagen, Denmark). The C2C12 cell line was obtained from Shanghai EK-Bioscience Biotechnology Co., Ltd. (Shanghai, China). C57BL/6J mice were purchased from Hangzhou Ziyuan Experimental Animal Technology Co., Ltd. (Hangzhou, China). Unless otherwise specified, analytical-grade reagents and commercial kits were purchased from Solarbio (Beijing, China). Unless otherwise specified, antibodies were purchased from HuaBio (Hangzhou, China).

### 2.2. Preparation of Pumpkin Seed Protein Peptides (PSPP)

Pumpkin seed protein powder was mixed with deionized water at a 1:8 (*w*/*w*) ratio and stirred until fully dissolved. The pH was adjusted to 8.5 using 2.0 M NaOH. After stabilization for 30 min, a compound protease preparation (Alcalase:flavor protease = 2:1, *w*/*w*) was added at 5.0% (*w*/*w*), and hydrolysis was conducted at 55 °C for 8 h. The reaction mixture was heated to 80 °C and maintained for 20 min to inactivate the enzymes. After cooling to room temperature, the mixture was centrifuged at 4 °C for 10 min. The supernatant was filtered through a 3 kDa molecular-weight-cutoff ultrafiltration membrane. The permeate was collected as pumpkin seed protein peptides (PSPP; protein content 92.30%, degree of hydrolysis 11.73%) and lyophilized for storage.

### 2.3. Cell Culture, Differentiation, and Viability Assay

C2C12 cells were cultured in growth medium (high-glucose DMEM supplemented with 10% fetal bovine serum, 100 U/mL penicillin, and 100 μg/mL streptomycin) at 37 °C in a humidified incubator with 5% CO_2_. At 80–90% confluence, the growth medium was replaced with differentiation medium (high-glucose DMEM supplemented with 2% horse serum, 100 U/mL penicillin, and 100 μg/mL streptomycin). The differentiation medium was refreshed every 2 days until myotube formation. Differentiated myotubes were treated with 20 μM DEX for 48 h to establish an in vitro sarcopenia model [29]. After DEX exposure, myotubes were incubated with PSPP (100, 200, 500, or 1000 μg/mL) for 72 h to evaluate the protective effect of PSPP against DEX-treated muscle atrophy. Cell viability was assessed using a CCK-8 assay to determine the PSPP concentration used in subsequent experiments.

### 2.4. SA-β-Gal Staining

After treatment, cells were washed with PBS and stained using a senescence-associated β-galactosidase (SA-β-gal) staining kit according to the manufacturer’s instructions. Images were acquired using a microscope, and the SA-β-gal-positive area was quantified using ImageJ Java v8.

### 2.5. ROS and Apoptosis Assays

After treatment, trypsinized cells were collected by centrifugation. Intracellular reactive oxygen species (ROS) levels and apoptosis were assessed using a ROS assay kit and an Annexin V-FITC apoptosis detection kit according to the manufacturer’s instructions. Flow cytometric analysis was performed on a CytoFLEX flow cytometer (Beckman Coulter, Inc., Brea, CA, USA), and data were analyzed using FlowJo software v10.8.1 (Becton, Dickinson and Company, Franklin Lakes, NJ, USA).

### 2.6. Myosin Heavy Chain (MyHC) Staining

After treatment, cells were fixed with 4% paraformaldehyde for 15 min and permeabilized with 0.5% Triton X-100 for 15 min at room temperature. Cells were blocked with 1% bovine serum albumin (BSA) for 2 h at room temperature and incubated with an anti-myosin heavy chain (MyHC) primary antibody overnight at 4 °C. After washing with PBS, cells were incubated with a FITC-labeled goat anti-rabbit IgG secondary antibody for 2 h in the dark. Nuclei were counterstained with 4′,6-diamidino-2-phenylindole (DAPI). Images were captured using a laser scanning confocal microscope, and the MyHC-positive area was quantified using ImageJ [30].

### 2.7. Total RNA Extraction and Real-Time qPCR Analysis

After treatment, total RNA was extracted from C2C12 cells using TRIzol reagent. RNA concentration and the A260/A280 ratio were measured using a microvolume spectrophotometer. Complementary DNA (cDNA) was synthesized using a reverse transcription kit. Quantitative PCR was performed using a real-time PCR system (SLAN-96S, Shanghai Hongshi Medical Technology Co., Ltd., Shanghai, China). β-Actin was used as the internal reference, and relative expression was calculated using the 2^−ΔΔCt^ method [31].

### 2.8. Western Blotting

After treatment, cells were washed with PBS. Proteins were extracted using radioimmunoprecipitation assay (RIPA) lysis buffer supplemented with phenylmethylsulfonyl fluoride (PMSF, 1:100, *v*/*v*), and protein concentration was determined using a BCA assay kit. Equal amounts of protein (20 μg per lane) were separated by 7.5% SDS-PAGE. Proteins were transferred to a polyvinylidene fluoride (PVDF) membrane and blocked with 3% BSA in tris-buffered saline with Tween 20 (TBST) at 37 °C for 90 min. The membrane was incubated overnight at 4 °C with primary antibodies against MuRF1 (1:1000), Atrogin-1 (1:2000), and MyoD (1:500). After washing with TBST, the membrane was incubated with a goat anti-rabbit IgG secondary antibody at 37 °C for 1 h. Protein bands were visualized using an enhanced chemiluminescence (ECL) substrate. Signals were captured using a gel imaging system (61006-9Q, Shanghai Qinxiang Scientific Instrument Co., Ltd., Shanghai, China). Band intensities were quantified using ImageJ and normalized to β-actin.

### 2.9. Animal Model, Administration, and Tissue Collection

Sixty-three 8-week-old male C57BL/6J mice were acclimated for 1 week under controlled conditions (20–25 °C, 50–60% humidity, 12 h light/12 h dark cycle) with ad libitum access to food and water. Mice were weighed and randomly assigned to seven groups: normal control (NC), DEX-treated (DEX), low-dose PSPP treatment (LT, 300 mg/kg/day), high-dose PSPP treatment (HT, 500 mg/kg/day), vitamin D treatment (DT, 3 μg/kg/day), low-dose combination vitamin D treatment (LDT, PSPP 300 mg/kg/day + vitamin D 3 μg/kg/day), and high-dose combination vitamin D treatment (HDT, PSPP 500 mg/kg/day + vitamin D 3 μg/kg/day). The dosage of PSPP was determined based on preliminary experiments and a review of relevant literature [17]. Except for the NC group, the sarcopenia model was established by intraperitoneal administration of DEX (25 mg/kg/day) for 10 consecutive days [6]. After model establishment, PSPP and/or vitamin D were administered orally for 30 days according to group assignment. Body weight and food intake were recorded every 10 days throughout the 40-day experimental period. At the end of the experiment, 150 μL of blood was collected via orbital enucleation, centrifuged for 10 min, and the serum was stored for subsequent analyses. Mice were euthanized by cervical dislocation. Hindlimb muscles, including the gastrocnemius, tibialis anterior, soleus, and extensor digitorum longus, were excised and weighed. The gastrocnemius and tibialis anterior indices were calculated as muscle weight (mg) divided by body weight (g). Samples for histopathological analysis were fixed in a universal tissue fixative.

### 2.10. Muscle Mass and Performance Tests

After the 30-day intervention, body composition was assessed using a small-animal body composition analyzer (QMR12-060H-1, Suzhou Niumag Analytical Instrument Co., Suzhou, China) to obtain lean mass and fat mass percentages. Grip strength was measured using a grip strength meter (XR501, Shanghai Xinruan Information Technology Co., Ltd., Shanghai, China), and endurance was evaluated using a rotarod fatigue test system (XR-6C/6D, Shanghai Xinruan Information Technology Co., Ltd., Shanghai, China). Measurements were recorded, and grip strength was normalized to body weight [20,32].

Relative grip strength (g/g) = grip strength (kgf)/body weight (g) × 1000 [33].

### 2.11. Histological Analysis

After fixation in a universal tissue fixative, gastrocnemius and tibialis anterior muscles were dehydrated, paraffin-embedded, sectioned at 4 μm, and subjected to hematoxylin and eosin (H&E) staining. Histological images were acquired using a microscope (Nikon Eclipse E100, Nikon Corporation, Tokyo, Japan). The mean cross-sectional area of muscle fibers in the gastrocnemius and tibialis anterior muscles was quantified using ImageJ software, with at least 100 fibers measured for each muscle.

### 2.12. ATP Content and mtDNA Quantification

Approximately 10 mg of gastrocnemius tissue was weighed and lysed in 100 μL of ATP lysis buffer. Samples were homogenized using a tissue grinder. After complete lysis, samples were centrifuged at 4 °C for 5 min, and the supernatant was collected. ATP content in gastrocnemius samples was measured using an ATP content assay kit according to the manufacturer’s instructions [32]. In parallel, mtDNA content in gastrocnemius muscle was estimated by quantifying the copy number of the mitochondrial-encoded gene ND1 using RT-qPCR.

### 2.13. Biochemical Measurements in Serum and Skeletal Muscle

Approximately 10 mg of gastrocnemius tissue was weighed and homogenized in 90 μL of PBS (0.01 M, pH 7.4) on ice. Homogenates were centrifuged at 4 °C for 10 min, and the supernatants were collected. Tumor necrosis factor-α (TNF-α), interleukin 10 (IL-10), malonic dialdehyde (MDA), and insulin-like growth factor-1 (IGF-1) levels in gastrocnemius homogenates and serum were determined using corresponding ELISA kits [16]. Serum 25-hydroxyvitamin D (25(OH)D) levels were measured according to the manufacturer’s instructions to reflect vitamin D nutritional status in mice.

### 2.14. Statistical Analysis

All experiments were performed at least three times, and results were presented as mean ± standard deviation (SD). One-way analysis of variance (ANOVA) followed by Tukey’s test was performed using SPSS 24 (SPSS Inc., Chicago, IL, USA). Differences were considered significant at *p* < 0.05.

## 3. Results and Discussion

### 3.1. PSPP Improves Cell Viability and Alleviates Senescence in DEX-Treated C2C12 Cells

**Figure 1:**
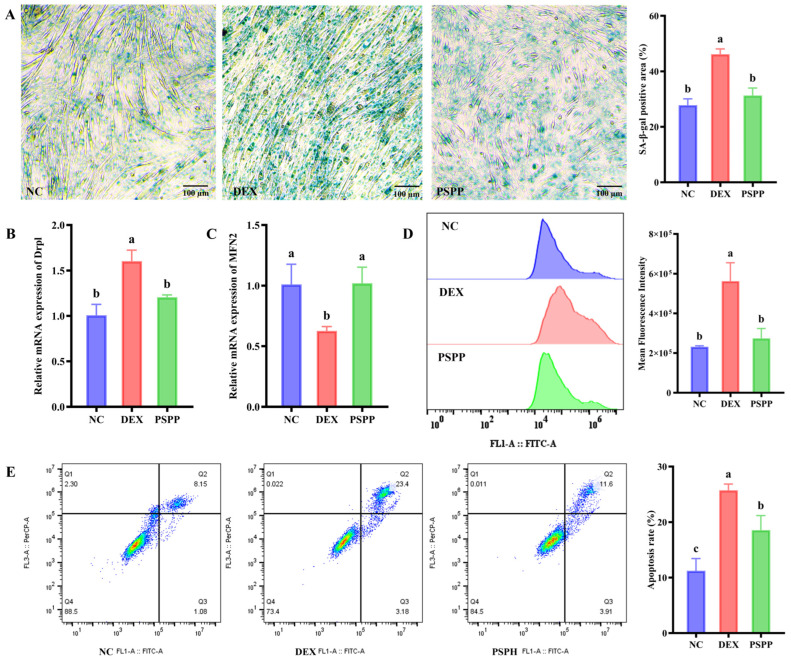
PSPP (500 μg/mL) attenuates DEX-treated injury in C2C12 cells. (**A**) SA-β-gal staining. (**B**,**C**) mRNA levels of Drp1 and MFN2. (**D**) Representative flow cytometry histograms and ROS levels. (**E**) Representative flow cytometry plots and apoptosis rates. Data are presented as mean ± SD (*n* = 3). Different lowercase letters above bars indicate significant differences among groups (*p* < 0.05; one-way ANOVA followed by Tukey’s test).

To evaluate the protective effects of PSPP in DEX-treated C2C12 cells, we assessed cell viability and senescence-associated markers. The CCK-8 assay (Figure S1) showed that 25 μM DEX reduced cell viability in the model group by 14%. PSPP at 100 μg/mL did not appreciably improve viability, whereas 200–1000 μg/mL PSPP restored viability to levels comparable to the control group (Figure S1). Based on these findings, 500 μg/mL PSPP was selected for subsequent experiments. SA-β-gal staining showed abundant positive cells in the model group, whereas PSPP reduced the SA-β-gal-positive area (Figure 1A), consistent with attenuation of DEX-treated senescence. Collectively, these data indicate that PSPP improved the viability of DEX-treated C2C12 cells and alleviated senescence-associated phenotypes.

### 3.2. PSPP Modulates Mitochondrial Homeostasis and Attenuates Oxidative Stress and Apoptosis

Dysregulated mitochondrial dynamics and oxidative stress have been implicated in sarcopenia progression [34]. To assess mitochondrial dynamics, we quantified Drp1 and MFN2 mRNA levels. Compared with the control group, DEX increased Drp1 mRNA (a fission-related factor) and decreased MFN2 mRNA (a fusion-related factor) (Figure 1B,C). These changes were consistent with a shift toward mitochondrial fission and may contribute to mitochondrial dysfunction, which has been linked to impaired muscle homeostasis, including altered mitophagy [35]. Mitochondrial impairment is often accompanied by excessive ROS accumulation, which can exacerbate oxidative stress and has been associated with activation of catabolic pathways (e.g., FoxO3a) and myofibrillar atrophy [36]. In contrast, PSPP partially reversed the DEX-treated changes in Drp1 and MFN2 and reduced ROS levels (Figure 1D). PSPP also reduced the proportions of early (Q3) and late (Q2) apoptotic cells, thereby decreasing the overall apoptosis rate (Figure 1E). Collectively, these findings suggest that PSPP may reduce DEX-treated cellular damage and apoptosis, potentially by modulating mitochondrial homeostasis and attenuating oxidative stress.

### 3.3. PSPP Attenuates Myofibrillar Atrophy by Suppressing Proteolysis and Supporting Myogenic Differentiation

**Figure 2:**
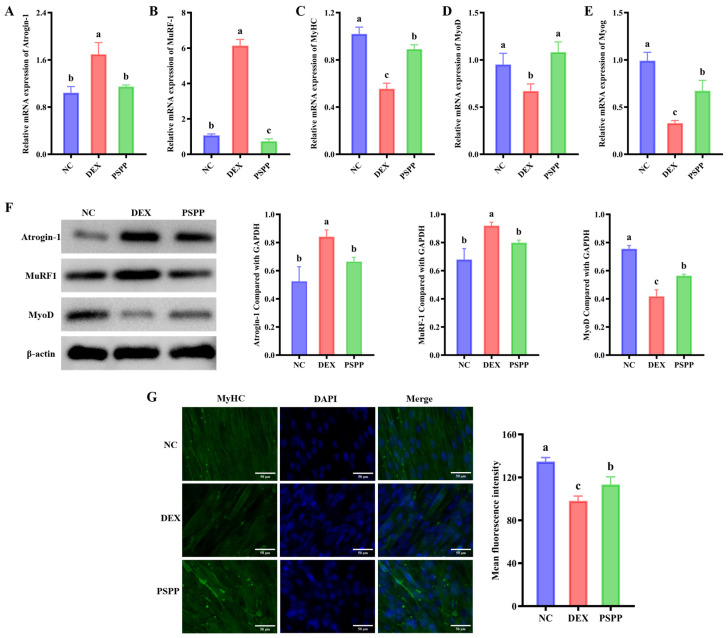
PSPP (500 μg/mL) attenuates DEX-treated myofibrillar atrophy. (**A**–**E**) mRNA levels of Atrogin-1, MuRF1, MyHC, MyoD, and Myog. (**F**) Representative Western blot images and Protein levels of Atrogin-1, MuRF1, and MyoD. (**G**) Representative immunofluorescence staining images and Myosin Heavy Chain Staining. Data are expressed as mean ± SD (*n* = 3). Different lowercase letters above bars indicate significant differences between groups (*p* < 0.05, one-way ANOVA followed by Tukey’s test).

The balance between muscle protein synthesis and degradation is critical for maintaining skeletal muscle mass, and ubiquitin-proteasome system (UPS)-mediated proteolysis is a major contributor to glucocorticoid-induced atrophy [37]. We used RT-qPCR and Western blotting to assess the effects of PSPP on atrophy-related genes and myogenic differentiation markers in C2C12 cells. RT-qPCR showed that DEX increased Atrogin-1 and MuRF1 mRNA levels (Figure 2A,B). Western blotting further showed increased Atrogin-1 and MuRF1 protein levels after DEX exposure (Figure 2F). PSPP attenuated DEX-treated increases in Atrogin-1 and MuRF1 and restored the mRNA levels of myogenic markers, including MyHC, MyoD, and Myog (Figure 2C–E). These changes were consistent with enhanced myogenic differentiation and myotube formation. Immunofluorescence analysis provided additional support for these observations. Myosin heavy chain (MyHC) is a widely used marker of myogenic differentiation and myotube formation. Compared to controls, DEX-treated C2C12 cells exhibited faint green fluorescence, reduced MyHC expression, disorganized myofibrillar structure, and impaired differentiation. PSPP intervention significantly increased immunofluorescent area and promoted more complete, continuous myotube structures (Figure 2G), consistent with improved myogenic capacity. Overall, the suppression of UPS-related markers (Atrogin-1 and MuRF1) and the restoration of myogenic markers (MyHC, MyoD, and Myog) suggest that PSPP may alleviate DEX-treated myofibrillar atrophy by attenuating proteolysis-related signaling and supporting myogenic differentiation.

### 3.4. PSPP and Vitamin D Restore Skeletal Muscle Mass in DEX-Treated Mice

**Figure 3:**
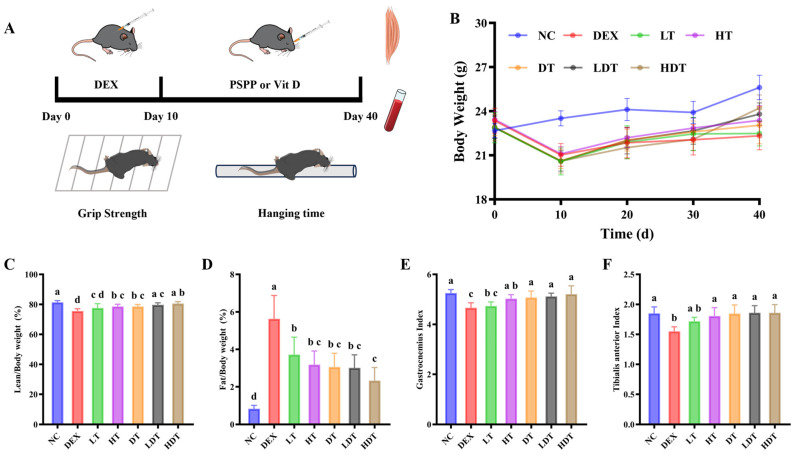
Effects of PSPP and vitamin D on body composition and skeletal muscle mass in DEX-treated mice. (**A**) Experimental timeline. (**B**) Changes in body weight. (**C**) Lean mass percentage. (**D**) Fat mass percentage. (**E**,**F**) Gastrocnemius and tibialis anterior muscle indices. Data are expressed as mean ± SD (*n* = 9). Different lowercase letters above bars indicate significant differences between groups (*p* < 0.05, one-way ANOVA followed by Tukey’s test). NC: normal control; DEX: DEX-treated model; LT: low-dose PSPP treatment; HT: high-dose PSPP treatment; DT: vitamin D treatment; LDT: low-dose PSPP combination vitamin D treatment; and HDT: high-dose PSPP combination vitamin D treatment.

**Figure 4:**
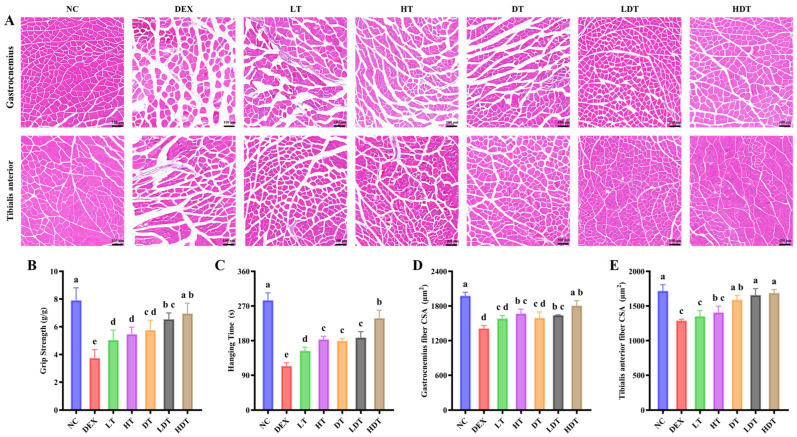
Effects of PSPP and vitamin D on myofiber morphology and muscle function in DEX-treated mice. (**A**) Representative H&E-stained sections of the gastrocnemius and tibialis anterior muscles (scale bar = 100 μm; 20×). (**B**) Grip strength. (**C**) Hanging time. (**D**,**E**) Cross-sectional area of muscle fibers in the gastrocnemius and tibialis anterior muscles. Data are expressed as mean ± SD (*n* = 9). Different lowercase letters above bars indicate significant differences between groups (*p* < 0.05, one-way ANOVA followed by Tukey’s test). NC: normal control; DEX: DEX-treated model; LT: low-dose PSPP treatment; HT: high-dose PSPP treatment; DT: vitamin D treatment; LDT: low-dose PSPP combination vitamin D treatment; and HDT: high-dose PSPP combination vitamin D treatment.

Although natural aging is considered the most appropriate model for sarcopenia, DEX-treated models are frequently used because of time and cost constraints [6]. To further investigate the effects of PSPP and vitamin D on muscle wasting, we induced muscle atrophy in mice via DEX administration. Compared with controls, 10 days of intraperitoneal DEX administration reduced body weight (Figure 3B). Dissection of hindlimb muscles showed that DEX reduced both the gastrocnemius and tibialis anterior indices (Figure 3E,F). Behavioral assessments further showed that DEX-treated mice exhibited reduced grip strength and endurance (Figure 4B,C), consistent with previous reports [38]. Food intake did not differ significantly among groups during the experimental period (Table S2), suggesting that the observed muscle and performance deficits were not attributable to reduced caloric intake. Following intervention, except for the low-dose PSPP group, DEX-treated mice showed varying degrees of improvement in body composition and skeletal muscle. Compared with the DEX group, body weight gradually increased in all intervention groups over the subsequent 30 days. PSPP at 500 mg/kg/day attenuated DEX-treated lean mass loss and fat accumulation (Figure 3C,D). As shown in Figure 3E,F and Figure S2, group differences in skeletal muscle weight and morphology were most evident in the gastrocnemius and tibialis anterior muscles. The gastrocnemius is commonly used as a representative skeletal muscle because of its large mass and its contribution to lower-limb force generation and stability [39]. Supplementation with high-dose PSPP or vitamin D increased the relative weights of both the gastrocnemius and tibialis anterior muscles, indicating reduced muscle loss; however, the effect of low-dose PSPP alone was comparatively modest. This difference may relate to the relatively short intervention period in the present study, as intervention durations exceeded eight weeks in similar studies [17,18].

### 3.5. PSPP and Vitamin D Restore Muscle Function and Improve Myofiber Morphology

Muscle fiber cross-sectional area and density, together with muscle strength and endurance, are commonly used indices of muscle mass and function. To assess group differences in muscle structure, we performed H&E staining of the gastrocnemius and tibialis anterior (TA) muscles (Figure 4A). Consistent with reduced muscle mass, DEX-treated mice showed marked myofiber degeneration and loosening, increased connective tissue, and inflammatory cell infiltration, indicating impaired tissue integrity. The low-dose intervention partially alleviated DEX-treated myofiber atrophy; however, fiber size remained heterogeneous, with numerous small fibers. With increasing PSPP dose, muscle fiber diameter and cross-sectional area increased, with more regular morphology. Under combined PSPP and vitamin D intervention, muscle fibers appeared more tightly arranged, with improved uniformity and structural integrity, and fiber cross-sectional area approached that of the control group (Figure 4D,E). These structural improvements may provide a morphological basis for enhanced muscle performance, as larger cross-sectional area and preserved fiber architecture are generally associated with greater force-generating capacity. To further evaluate functional outcomes, muscle strength and endurance/coordination were assessed using a grip strength meter and a rotarod fatigue test, respectively (Figure 4B,C). At 300 mg/kg/day, PSPP partially improved DEX-treated motor dysfunction, with increased grip strength and hanging time. However, reduced force sustainability, susceptibility to fatigue, and impaired coordination persisted during testing. High-dose PSPP (500 mg/kg/day) or vitamin D produced greater improvements in muscle mass and function. Vitamin D has been reported to influence muscle strength and mass through mechanisms involving inflammatory regulation, Ca^2+^ homeostasis, and mitochondrial stabilization [40]. The high-dose combined intervention improved muscle mass, functional performance, and muscle morphology in model mice and showed the greatest overall protective effect against DEX-treated muscle atrophy among the tested regimens. This outcome was consistent with a potential additive benefit of PSPP and vitamin D co-intervention.

### 3.6. PSPP and Vitamin D Improve Mitochondrial Function and Skeletal Muscle Metabolic Capacity

**Figure 5:**
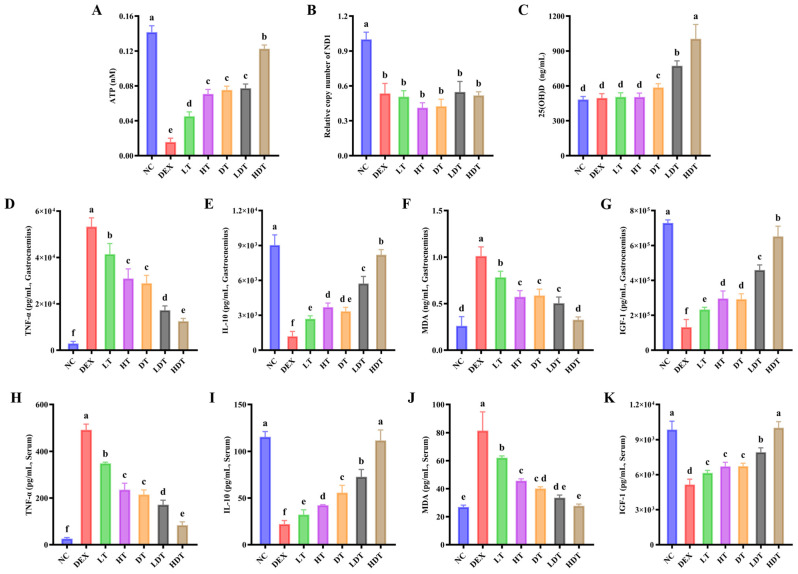
Effects of PSPP and vitamin D on mitochondrial function, inflammatory cytokines, and oxidative stress in DEX-treated mice. (**A**) ATP content. (**B**) Relative ND1 copy number. (**C**) Serum 25(OH)D level. (**D**–**G**) Levels of TNF-α, IL-10, IGF-1, and MDA in gastrocnemius muscle. (**H**–**K**) Serum levels of TNF-α, IL-10, IGF-1, and MDA. Data are expressed as mean ± SD (*n* = 9). Different lowercase letters above bars indicate significant differences between groups (*p* < 0.05, one-way ANOVA followed by Tukey’s test). NC: normal control; DEX: DEX-treated model; LT: low-dose PSPP treatment; HT: high-dose PSPP treatment; DT: vitamin D treatment; LDT: low-dose PSPP combination vitamin D treatment; and HDT: high-dose PSPP combination vitamin D treatment.

Movement is a primary physiological function of skeletal muscle. ATP generated through mitochondrial respiration supplies the energy required for rapid muscle contraction and relaxation. Reduced mitochondrial efficiency can limit ATP production, and ATP content in the gastrocnemius muscle reflects, at least in part, mitochondrial ATP-producing capacity. We measured total ATP content in gastrocnemius muscle across groups. As shown in Figure 5A, DEX exposure was associated with impaired mitochondrial oxidative metabolism and reduced gastrocnemius ATP content to 0.015 nM. Compared with model mice, both PSPP and vitamin D increased ATP content in the gastrocnemius muscle, suggesting improved energetic capacity under intervention. These results suggest that both interventions may improve mitochondrial ATP-producing capacity in gastrocnemius muscle, thereby helping meet the energetic demands of movement. This may partly explain the improvements in grip strength and hanging time observed in intervention groups. We further estimated mitochondrial abundance by measuring relative mtDNA copy number in gastrocnemius muscle. Mitochondrial abundance can influence ATP content in muscle. As shown in Figure 5B, DEX reduced mtDNA copy number in gastrocnemius muscle by approximately 50%. However, PSPP and vitamin D did not markedly reverse the DEX-treated reduction in mtDNA copy number (Figure 5B). In summary, the increase in ATP content did not appear to be driven by increased mitochondrial abundance and may instead reflect improved mitochondrial homeostasis and efficiency.

### 3.7. PSPP and Vitamin D Attenuate Inflammation and Oxidative Stress in DEX-Treated Mice

Excessive pro-inflammatory cytokines are negatively associated with skeletal muscle mass, strength, and function. These cytokines can activate signaling pathways that disrupt the balance between muscle protein synthesis and degradation and reduce IGF-1 levels, thereby contributing to sarcopenia progression. TNF-α, a key inflammatory mediator in muscle atrophy, has been reported to promote proteolysis and suppress myogenic regulators (e.g., MyoD and MyoG), which can exacerbate muscle loss; elevated TNF-α levels have been associated with an increased risk of sarcopenia [41,42]. In this study, PSPP reduced pro-inflammatory markers in both muscle and serum. Following high-dose PSPP, TNF-α decreased by 28% in muscle and 52% in serum, whereas IL-10 increased, suggesting partial correction of the inflammatory imbalance in DEX-treated mice. Muscle atrophy is often accompanied by heightened oxidative stress, which may further amplify inflammatory responses. Oxidative stress was assessed by measuring MDA levels. PSPP reduced MDA levels in both muscle and serum in a dose-dependent manner. Additionally, vitamin D showed anti-inflammatory and antioxidant effects comparable to high-dose PSPP. Consistently, high-dose PSPP combined with vitamin D produced the most pronounced overall improvement, with IL-10 and MDA levels approaching those of the control group. This pattern aligned with the observed recovery of muscle mass and function and may involve improved vitamin D bioavailability or utilization (Figure 5C). Furthermore, IGF-1 is a key anabolic signal involved in muscle protein synthesis and myogenic differentiation, and it supports skeletal muscle development and function in part through growth hormone-related signaling [43]. PSPP and vitamin D increased IGF-1 levels, further supporting the protective effects of each treatment against DEX-treated muscle atrophy in mice.

## 4. Conclusions

This study evaluated the effects of PSPP, alone or combined with vitamin D, in DEX-treated sarcopenia models in C2C12 cells and mice. In vitro, PSPP attenuated DEX-treated cellular injury by reducing senescence, oxidative stress, and apoptosis, while suppressing ubiquitin–proteasome-associated atrophy markers (Atrogin-1 and MuRF1) and restoring myogenic differentiation markers (MyHC, MyoD, and Myog). In vivo, high-dose PSPP, vitamin D, and their combination improved DEX-treated declines in gastrocnemius and tibialis anterior muscle mass and functional performance in mice, with the high-dose co-intervention showing the greatest overall benefit among the tested regimens. These improvements were accompanied by increased ATP content, reduced TNF-α and MDA in muscle and/or serum, and increased IL-10 and IGF-1. Integrating the cellular and animal findings, the protective pattern observed in this study may involve IGF-1-linked anabolic signaling, mitochondrial homeostasis/efficiency, redox balance, and inflammatory modulation. Nonetheless, the in vivo analyses primarily emphasized functional and histological outcomes, and additional mechanistic studies will be required to delineate the underlying molecular pathways. Although DEX-treated models offer a practical option for preclinical research, and glucocorticoid-induced and naturally aged mouse models show similar changes in body composition and muscle function, age-related sarcopenia involves complex molecular mechanisms, making it difficult to fully recapitulate in vivo conditions. Overall, these findings position PSPP as a food-derived bioactive peptide candidate and support PSPP-vitamin D co-intervention as a promising nutritional strategy to mitigate glucocorticoid-associated muscle atrophy phenotypes in preclinical models.

## Data Availability

The original contributions presented in this study are included in the article/Supplementary Material. Further inquiries can be directed to the corresponding authors.

## Supplementary Materials

The following supporting information can be downloaded at: https://www.mdpi.com/article/10.3390/foods15071162/s1, Figure S1: Effects of PSPP on C2C12 cell viability. Data are expressed as mean ± SD (*n* = 3). Different letters above bars indicate significant differences between groups (*p* < 0.05, one-way ANOVA followed by Tukey’s test), Figure S2: Representative images of the gastrocnemius (Gast), tibialis anterior (TA), soleus (Sol), and extensor digitorum longus (ESL) muscles, Table S1: Molecular weight distribution of PSPP (%), Table S2: Changes in food intake during the experiment (g).

## Abbreviations

The following abbreviations are used in this manuscript:

PSPP	Pumpkin seed protein peptides
DEX	Dexamethasone
IGF-1	Insulin-like growth factor-1
TNF-α	Tumor necrosis factor-α
IL-10	Interleukin 10
MDA	Malonic dialdehyde
25(OH)D	25-hydroxyvitamin D

## References

[1] Tieland M., Trouwborst I., Clark B.C. (2018). Skeletal muscle performance and ageing. J. Cachexia Sarcopenia Muscle.

[2] Yuan S., Larsson S.C. (2023). Epidemiology of sarcopenia: Prevalence, risk factors, and consequences. Metabolism.

[3] Cruz-Jentoft A.J., Bahat G., Bauer J., Boirie Y., Bruyère O., Cederholm T., Cooper C., Landi F., Rolland Y., Sayer A.A. (2019). Sarcopenia: Revised European consensus on definition and diagnosis. Age Ageing.

[4] Papadopoulou S.K., Tsintavis P., Potsaki G., Papandreou D. (2020). Differences in the Prevalence of Sarcopenia in Community-Dwelling, Nursing Home and Hospitalized Individuals. A Systematic Review and Meta-Analysis. J. Nutr. Health Aging.

[5] Larsson L., Degens H., Li M., Salviati L., Lee Y.I., Thompson W., Kirkland J.L., Sandri M. (2019). Sarcopenia: Aging-Related Loss of Muscle Mass and Function. Physiol. Rev..

[6] Wang B.Y., Hsiao A.W., Wong N., Chen Y.F., Lee C.W., Lee W.Y.W. (2023). Is dexamethasone-induced muscle atrophy an alternative model for naturally aged sarcopenia model?. J. Orthop. Transl..

[7] Calcaterra L., Abellan van Kan G., Steinmeyer Z., Angioni D., Proietti M., Sourdet S. (2024). Sarcopenia and poor nutritional status in older adults. Clin. Nutr..

[8] Files D.C., Liu C., Pereyra A., Wang Z.M., Aggarwal N.R., D’Alessio F.R., Garibaldi B.T., Mock J.R., Singer B.D., Feng X. (2015). Therapeutic exercise attenuates neutrophilic lung injury and skeletal muscle wasting. Sci. Transl. Med..

[9] Yin L., Li N., Jia W., Wang N., Liang M., Shang J., Qiang G., Du G., Yang X. (2022). Urotensin receptor acts as a novel target for ameliorating fasting-induced skeletal muscle atrophy. Pharmacol. Res..

[10] Lee J.Y., Shin S.K., Bae H.R., Ji Y., Park H.J., Kwon E.Y. (2023). The animal protein hydrolysate attenuates sarcopenia via the muscle-gut axis in aged mice. Biomed. Pharmacother..

[11] Wu Y., de Crom T.O.E., Chen Z., Benz E., van der Schaft N., Pinel A., Boirie Y., Eglseer D., Topinkova E., Schoufour J.D. (2025). Dietary protein intake and body composition, sarcopenia and sarcopenic obesity: A prospective population-based study. Clin. Nutr..

[12] Chang M.C., Choo Y.J. (2023). Effects of Whey Protein, Leucine, and Vitamin D Supplementation in Patients with Sarcopenia: A Systematic Review and Meta-Analysis. Nutrients.

[13] Qiu J., Cheng Y., Deng Y., Ren G., Wang J. (2023). Composition of gut microbiota involved in alleviation of dexamethasone-induced muscle atrophy by whey protein. NPJ Sci. Food.

[14] Hyun J., Kim G., Park S., Kim J., Kim J.-I., Ryu B. (2025). Amelioration of muscle wasting by red algal protein: The preventive potential of Pyropia dentata protein In vitro and In vivo study. Food Biosci..

[15] Li C., Meng H., Wu S., Fang A., Liao G., Tan X., Chen P., Wang X., Chen S., Zhu H. (2021). Daily Supplementation with Whey, Soy, or Whey-Soy Blended Protein for 6 Months Maintained Lean Muscle Mass and Physical Performance in Older Adults with Low Lean Mass. J. Acad. Nutr. Diet..

[16] Pan D., Yang L., Yang X., Xu D., Wang S., Gao H., Liu H., Xia H., Yang C., Lu Y. (2024). Potential nutritional strategies to prevent and reverse sarcopenia in aging process: Role of fish oil-derived ω-3 polyunsaturated fatty acids, wheat oligopeptide and their combined intervention. J. Adv. Res..

[17] Guo D., Zou H., Chen M., Wei S., Cai Y., Wang Z., Wang H., Yi Y., Xu W. (2025). Corn-derived peptide LQQQLL alleviates skeletal muscle attenuation by mTOR signaling pathway and intestinal microbiota. Food Res. Int..

[18] Chai H.-J., Yi T.-K., Felim J., Tu M.-C., Kong Z.-L. (2025). Synergistic effects of fucoidan from Undaria pinnatifida and grouper peptides on glucocorticoid-induced muscle atrophy via protein turnover modulation. Biomed. Pharmacother..

[19] Lin C., Zeng J., Zhang S., Xu X., Chen L., Yang Z., Wu W., Hu C., Zhao Y.-T. (2024). Remedial effects of tilapia skin peptides against dexamethasone-induced muscle atrophy in mice by modulation of AKT/FOXO3a and Sirt1/PGC-1α signaling pathways. J. Funct. Foods.

[20] Kim S.-M., Kim J.-Y., Jun E.-M., Jaiswal V., Park E.-J., Lee H.-J. (2025). Mealworm hydrolysate ameliorates dexamethasone-induced muscle atrophy via sirtuin 1-mediated signaling and Akt pathway. NPJ Sci. Food.

[21] Hwangbo Y., Pan J.H., Lee J.J., Kim T., Kim J.H. (2024). Production of protein hydrolysates from hemp (*Cannabis sativa* L.) seed and its protective effects against dexamethasone-induced muscle atrophy. Food Biosci..

[22] Vinayashree S., Vasu P. (2021). Biochemical, nutritional and functional properties of protein isolate and fractions from pumpkin (*Cucurbita moschata* var. Kashi Harit) seeds. Food Chem..

[23] Coelho Pacheco A.F., Coelho Pacheco F., Silva Cunha J., Ribeiro de Santos F., Coelho Pacheco J.C., de Paula Correa K., de Almeida Orlando Junior W., Costa Paiva P.H., de Castro Leite Junior B.R. (2024). Bibliometric analysis of pumpkin seed proteins: A review of the multifunctional properties of their hydrolysates and future perspectives. Food Biosci..

[24] Rong L., Ouyang K., Liu M., Xiao F., Chen Y., Woo M.W., Zhao Q. (2025). Valorizing pumpkin (*Cucurbita moschata*) seed meal into protein hydrolysates: Impact of different proteases on the structural, physicochemical, and functional properties. Food Biosci..

[25] Habib M., Singh S., Hanan E., Jan K., Bashir K. (2025). Optimization of enzymatic hydrolysis for obtaining antioxidant hydrolysates from pumpkin seed protein: Improvement of the physicochemical, structural and functional properties. Appl. Food Res..

[26] Prokopidis K., Testa G.D., Giannaki C.D., Stavrinou P., Kelaiditi E., Hoogendijk E.O., Veronese N. (2025). Prognostic and Associative Significance of Malnutrition in Sarcopenia: A Systematic Review and Meta-Analysis. Adv. Nutr..

[27] Salles J., Chanet A., Guillet C., Vaes A.M., Brouwer-Brolsma E.M., Rocher C., Giraudet C., Patrac V., Meugnier E., Montaurier C. (2022). Vitamin D status modulates mitochondrial oxidative capacities in skeletal muscle: Role in sarcopenia. Commun. Biol..

[28] Vaes A.M.M., Tieland M., Toussaint N., Nilwik R., Verdijk L.B., van Loon L.J.C., de Groot L. (2018). Cholecalciferol or 25-Hydroxycholecalciferol Supplementation Does Not Affect Muscle Strength and Physical Performance in Prefrail and Frail Older Adults. J. Nutr..

[29] He Y., Tan Y., Song Z., Chen Y. (2025). Ferulic acid attenuates Sarcopenia progression by inhibiting peroxisomal ACOX1. Free Radic. Biol. Med..

[30] Kim J.W., Yun S., Park M.J., Song E., Jang S., Jang A., Choi K.M., Baik S.H., Hwang H.J., Yoo H.J. (2025). HD6277 Suppresses Muscle Atrophy by Promoting Myogenic Factors and Inhibiting Proteolysis in Aged Mice. J. Cachexia Sarcopenia Muscle.

[31] Zhang Z., Fang Y., He Y., Farag M.A., Zeng M., Sun Y., Peng S., Jiang S., Zhang X., Chen K. (2024). Bifidobacterium animalis Probio-M8 improves sarcopenia physical performance by mitigating creatine restrictions imposed by microbial metabolites. npj Biofilms Microbiomes.

[32] Chen W., Shen Z., Dong W., Huang G., Yu D., Chen W., Yan X., Yu Z. (2024). Polygonatum sibiricum polysaccharide ameliorates skeletal muscle aging via mitochondria-associated membrane-mediated calcium homeostasis regulation. Phytomedicine.

[33] Zheng Y., An S., Kim G.-Y., Han K.-S., Baek Y.-J., Kang I.-J. (2024). Glycyrrhiza uralensis extract improves dexamethasone-induced sarcopenia via regulating myostatin- and FoxO3a-mediated E3 ubiquitin ligase. Food Biosci..

[34] Zhang H., Qi G., Wang K., Yang J., Shen Y., Yang X., Chen X., Yao X., Gu X., Qi L. (2023). Oxidative stress: Roles in skeletal muscle atrophy. Biochem. Pharmacol..

[35] Lee T.T., Chen P.L., Su M.P., Li J.C., Chang Y.W., Liu R.W., Juan H.F., Yang J.M., Chan S.P., Tsai Y.C. (2021). Loss of Fis1 impairs proteostasis during skeletal muscle aging in Drosophila. Aging Cell.

[36] Xu X., Pang Y., Fan X. (2025). Mitochondria in oxidative stress, inflammation and aging: From mechanisms to therapeutic advances. Signal Transduct. Target. Ther..

[37] Sartori R., Romanello V., Sandri M. (2021). Mechanisms of muscle atrophy and hypertrophy: Implications in health and disease. Nat. Commun..

[38] Bae S., Mai V.H., Mun S., Dong D., Han K., Park S., Hyun J.K. (2025). Lonafarnib Protects Against Muscle Atrophy Induced by Dexamethasone. J. Cachexia Sarcopenia Muscle.

[39] Sebastián D., Beltrà M., Irazoki A., Sala D., Aparicio P., Aris C., Alibakhshi E., Rubio-Valera M., Palacín M., Castellanos J. (2024). TP53INP2-dependent activation of muscle autophagy ameliorates sarcopenia and promotes healthy aging. Autophagy.

[40] Wu D., Hu Q., Tan B., Rose P., Zhu D., Zhu Y.Z. (2018). Amelioration of mitochondrial dysfunction in heart failure through S-sulfhydration of Ca^2+^/calmodulin-dependent protein kinase II. Redox Biol..

[41] Tuttle C.S.L., Thang L.A.N., Maier A.B. (2020). Markers of inflammation and their association with muscle strength and mass: A systematic review and meta-analysis. Ageing Res. Rev..

[42] Rom O., Reznick A.Z. (2016). The role of E3 ubiquitin-ligases MuRF-1 and MAFbx in loss of skeletal muscle mass. Free Radic. Biol. Med..

[43] Li H., Wang R., Wang L., Li L., Ma Y., Zhou S. (2021). Bovine Milk Fat Globule Epidermal Growth Factor VIII activates PI3K/Akt signaling pathway and attenuates sarcopenia in rat model induced by d-galactose. Food Biosci..

