# Supplementary material for: Ameliorative Effects of Pumpkin Seed Protein Peptides on Dexamethasone-Treated Sarcopenia and Their Effects When Combined with Vitamin D
Source: Foods. 2026 Mar 30;15(7):1162. doi: 10.3390/foods15071162 (PMC13074051; doi:10.3390/foods15071162)
Supplement: Supplementary file 1 [file foods-15-01162-s001.zip › foods-4174339-supplementary.pdf]

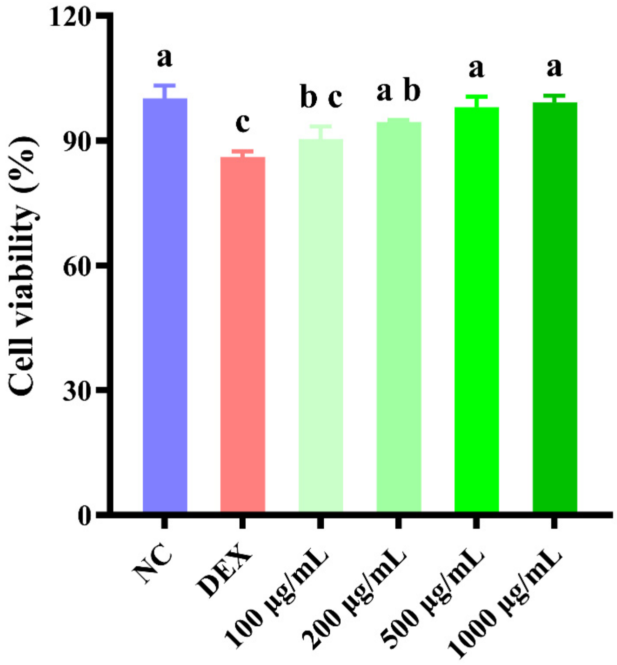

Figure S1. Effects of PSPP on C2C12 cell viability. Data are expressed as mean ± SD (n =3). Different letters above bars indicate significant differences between groups ($p < 0.05$, one-way ANOVA followed by Tukey's test).

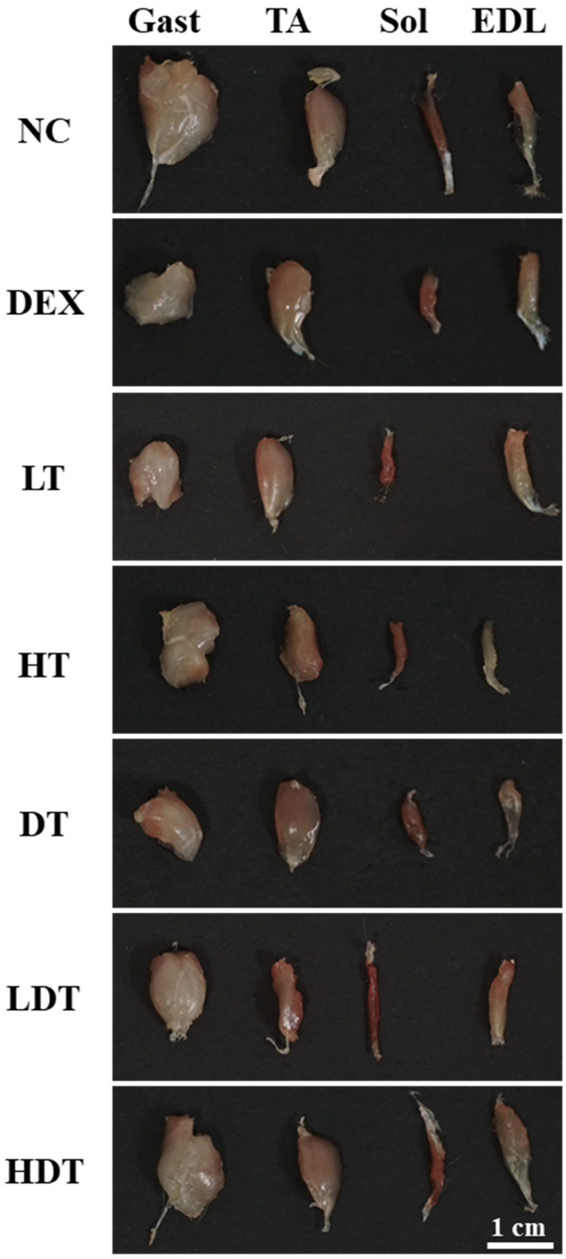

Figure S2. Representative images of the gastrocnemius (Gast), tibialis anterior (TA), soleus (Sol), and extensor digitorum longus (ESL) muscles.

Table S1. Molecular weight distribution of PSPP (%)

Molecular weight (Da)	PSPP
＞10000	4.10 ± 0.39
5000-3000	6.64 ± 0.68
3000-2000	7.94 ± 0.40
2000-1000	17.27± 1.47
＜1000	64.06 ± 4.95

Table S2. Changes in food intake during the experiment (g).

Group	10 d	20 d	30 d	40 d
NC	38.00	37.12	39.86	39.43
DEX	37.70	35.23	36.98	36.88
LT	38.20	36.20	36.02	37.34
HT	36.90	36.32	35.73	37.18
DT	38.10	37.78	37.32	37.96
LDT	37.50	36.34	37.49	37.96
HDT	38.40	36.45	38.73	37.25
